# Incidence, Treatment Patterns, and Associated Clinical Conditions of Hyperprolactinemia Identified via Nationwide Claims Data in Korea: A 13-Year Population-Based Study

**DOI:** 10.3390/jcm15124411

**Published:** 2026-06-07

**Authors:** Hyonjee Yoon, Kyung-Hee Chae, Hyunkyung Kim, Youngseo Jang, Chaewon Kim, Sukil Kim, Jeong Namkung

**Affiliations:** 1 Department of Obstetrics and Gynecology, Eunpyeong St. Mary’s Hospital, College of Medicine, The Catholic University of Korea, Seoul 06591, Republic of Korea; hjee6@naver.com (H.Y.); 95little@naver.com (Y.J.);; 2 Department of Preventive Medicine, College of Medicine, The Catholic University of Korea, Seoul 06591, Republic of Korea; 3 Department of Obstetrics and Gynecology, Uijeongbu St. Mary’s Hospital, College of Medicine, The Catholic University of Korea, Seoul 06591, Republic of Korea

**Keywords:** hyperprolactinemia, prevalence, incidence, cabergoline, bromocriptine

## Abstract

**Background:** Hyperprolactinemia is a common endocrine disorder with significant reproductive and systemic implications. This study aimed to investigate the nationwide epidemiological trends, longitudinal shifts in pharmacological treatment, and the temporal associations of concurrent conditions and long-term sequelae in Korean women with claims-based hyperprolactinemia. **Methods:** A nationwide, population-based retrospective cohort study was conducted using data from the Health Insurance Review & Assessment Service (HIRA) of South Korea from 2009 to 2021. Female patients aged 10–59 years with hyperprolactinemia diagnostic claims were evaluated. We analyzed annual prevalence, incidence, diagnostic procedures, and dopamine agonist prescription patterns. Associated clinical conditions were classified into two categories based on the timing of their diagnosis relative to hyperprolactinemia: concurrent or underlying conditions present at baseline, and long-term complications that developed during the follow-up period. **Results:** A total of 95,616 female patients were identified after applying the selection criteria. The prevalence and incidence of hyperprolactinemia peaked among women in their early thirties, with an absolute peak at age 32. A significant pharmacological paradigm shift was observed: bromocriptine was the predominant therapy during the early study period, but cabergoline prescriptions surpassed bromocriptine in 2017. Regarding clinical work-ups, only 5.3% of the entire cohort underwent a sella magnetic resonance imaging (MRI). Regarding associated clinical conditions, reproductive disorders such as infertility (28.0%) and polycystic ovary syndrome (24.8%) showed high overall prevalence but low incidence of new diagnoses during the follow-up period. Conversely, among the patients affected by bone disorders, more than 60% of the total osteoporosis and osteopenia cases were diagnosed subsequent to the initial hyperprolactinemia diagnosis. Significant post-diagnosis incidence was also observed for metabolic disorders, including dyslipidemia and diabetes mellitus. **Conclusions:** Hyperprolactinemia in Korean women is highly concentrated in the peak reproductive years. The shift toward cabergoline reflects evolving clinical guidelines and improved drug accessibility. Our findings highlight that while reproductive issues often present concurrently, bone loss and metabolic complications frequently emerge as post-diagnosis sequelae. Therefore, clinical management should extend beyond prolactin normalization to include proactive, multidisciplinary screening for skeletal and metabolic health.

## 1. Introduction

Hyperprolactinemia is one of the most common endocrine conditions of the hypothalamic–pituitary axis, characterized by persistently elevated serum prolactin levels [1,2]. In women of reproductive age, the condition predominantly manifests with clinical symptoms such as amenorrhea, oligomenorrhea, galactorrhea, and infertility [2]. These symptoms are primarily driven by prolactin-induced suppression of gonadotropin-releasing hormone (GnRH) pulsatility, which consequently leads to hypogonadism and estrogen deficiency [1,3].

The etiology of hyperprolactinemia is highly diverse, ranging from organic, tumor-related causes to functional and pharmacological factors [1,2,4,5]. Prolactin-producing pituitary adenomas (prolactinomas), or mixed co-secreting tumors such as somatotropinomas, represent the primary organic drivers of this condition [6,7]. Conversely, non-tumor-related or functional hyperprolactinemia can arise from secondary factors, including systemic conditions like hypothyroidism [8] or chronic kidney disease [9], as well as overlapping clinical syndromes such as polycystic ovary syndrome (PCOS) [4,10,11,12]. Additionally, it can be drug-induced, stemming from medications that disrupt dopaminergic pathways [13].

The cornerstone of medical management for hyperprolactinemia is pharmacological therapy using dopamine agonists, with bromocriptine and cabergoline being the most widely utilized agents [7,14]. In recent years, clinical guidelines have increasingly favored cabergoline due to its higher efficacy in normalizing prolactin levels and reducing pituitary tumor size, as well as its superior tolerability and patient compliance [7,15]. The clinical objective of this pharmacological intervention varies depending on the underlying etiology; it is utilized either as a primary therapy directly targeting the tumor mass in organic cases, or as an adjuvant treatment within specialized protocols for functional hyperprolactinemia, PCOS, and infertility [16,17]. Despite these clinical recommendations, comprehensive real-world data illustrating the longitudinal shifts and transitions in prescription patterns at a national population level remain scarce.

Furthermore, hyperprolactinemia is clinically intertwined with various systemic conditions through distinct molecular pathways. Patho-physiologically, it impairs reproduction by suppressing hypothalamic kisspeptin-1 and GnRH pulsatility, leading to infertility and anovulatory patterns that frequently overlap with PCOS [10,17,18,19,20]. Chronically elevated prolactin also drives metabolic changes; prolactin receptor activation in adipose tissue and pancreatic beta-cells worsens insulin resistance and reduces adiponectin, accelerating obesity and metabolic syndrome [21,22,23,24]. Moreover, the resulting hypogonadism and estrogen deficiency favor osteoclastogenesis, leading to long-term skeletal risks such as osteopenia and osteoporosis [25]. While these pathophysiological mechanisms are well-established, large-scale, population-based epidemiological data on its development and incidence in clinical practice remain limited.

Therefore, this study aimed to investigate the epidemiological characteristics, including the nationwide prevalence and incidence, of hyperprolactinemia among Korean women using a comprehensive healthcare claims database. We also sought to evaluate the longitudinal paradigm shifts in pharmacological treatment patterns over a 13-year period and to delineate the temporal associations between hyperprolactinemia and its major associated clinical conditions.

## 2. Materials and Methods

### 2.1. Study Design and Data Source

This nationwide, population-based retrospective cohort study was conducted using customized research data provided by the Health Insurance Review & Assessment Service (HIRA) of South Korea. The HIRA database contains comprehensive healthcare claims data for the entire South Korean population, including demographic information, diagnostic codes based on the International Classification of Diseases (ICD-10), and detailed records of outpatient visits, inpatient care, procedures, and prescriptions. The data is open for use for research through anonymization and deidentification of the subjects.

### 2.2. Case Definition and Study Population

We initially identified 115,906 female patients who had been diagnosed with hyperprolactinemia (ICD-10 code E221). To establish an incident cohort, a look-back period was applied to exclude patients with a pre-existing diagnosis of hyperprolactinemia between 2007 and 2008. Patients with a concurrent diagnosis of Parkinson’s disease (ICD-10 code G21) were excluded to eliminate the potential confounding effects of high-dose dopamine agonist therapy. Additionally, patients aged 9 years or younger and 60 years or older were excluded to focus comprehensively on women across the entire endocrine lifespan, from adolescence through postmenopause. Specifically, the inclusive age span of 10–59 years was selected, allowing us to capture early-adolescent cases arising during pubertal transitions or from drug-induced etiologies [26], as well as perimenopausal and postmenopausal presentations where hyperprolactinemia predominantly manifests as chronic skeletal of metabolic complications [27,28].

### 2.3. Calculation of Prevalence and Incidence

To estimate the annual prevalence of hyperprolactinemia, the total number of patients who met the case definition criteria was divided by the total number of all female population aged 10–59 years for each year. Incidence was defined as the first appearance of diagnostic codes for hyperprolactinemia in health insurance claims, regardless of hospital admissions or outpatient visits. A one-year look-back period method was applied to evaluate the estimated incidence to exclude cases with pre-existing diagnoses, thereby accurately distinguishing newly diagnosed incident cases from prevalent cases [29]. Both crude prevalence and incidence rates were expressed as the number of cases per 100,000 persons. Additionally, age-adjusted prevalence rates were calculated utilizing the direct standardization method, using the 2007 study cohort as the standard female population.

### 2.4. Assessment of Diagnostic and Treatment Patterns

Clinical assessment patterns were categorized into two groups: etiological diagnostic evaluations and cardiovascular safety surveillance. Etiological diagnostic evaluations, aimed at identifying underlying structural sellar lesions (such as prolactinoma), were tracked via codes for sella magnetic resonance imaging (MRI) and sella cone view radiography. Of note, sella cone view radiography represents a historical plain-film modality that is not part of modern diagnostic guidelines, but was captured in our dataset to reflect real-world clinical documentation spanning ack to 2009. Cardiovascular safety and complication surveillance—relevant to monitoring potential valvular hear disease from long-term, high-dose cabergoline therapy or secondary cardiovascular risks from prolonged untreated hypogonadism—was assessed using claims codes for electrocardiography (ECG) and echocardiography (transthoracic or transesophageal).

Pharmacological treatment patterns were evaluated based on the prescription records of dopamine agonists, specifically bromocriptine and cabergoline. Prescription distributions were analyzed by age group, and longitudinal trends in the annual prescription volume were tracked to assess shifts in clinical practice.

### 2.5. Definition of Associated Clinical Conditions and Temporal Associations

A comprehensive analysis of associated clinical conditions was conducted using ICD-10 codes, classified into four main categories: (1) endocrine and metabolic disorders (diabetes mellitus; E10-E14, thyroid disease; E00-E07, dyslipidemia; E78, obesity; E65-E68, and hypertension; I10-I15), (2) gynecological and reproductive disorders (infertility; N97.0-N97.9, polycystic ovary syndrome; E28.2, (3) bone disorders (osteoporosis; M80-M82, and osteopenia; M85.89), and (4) malignancies (breast cancer; C50, vulvar cancer; C51, vaginal cancer; C52, cervical cancer; C53, endometrial cancer; C54.1, ovarian cancer; C56, and other gynecological cancers; C57). To evaluate the temporal relationship between hyperprolactinemia and these associated conditions, the analysis was distinguished into two metrics: (1) overall prevalence, defined as the proportion of the hyperprolactinemia cohort who had the clinical condition at any point, and (2) post-diagnosis incidence, strictly defined as conditions that were newly diagnosed after the initial diagnosis of hyperprolactinemia. Post-diagnosis incidence was strictly tracked from each patient’s index hyperprolactinemia diagnosis date until 31 December 2021, allowing a longitudinal observation window of up to 13 years.

### 2.6. Statistical Analyses

All descriptive statistics were reported as numbers and percentages. Categorical variables were expressed as frequencies and proportions. A 95% binomial confidence interval (CI) was initially considered for estimating the prevalence and incidence. However, owing to the exceptionally large population size of this cohort, the standard errors were negligibly small, resulting in values that were virtually identical to the point estimates for all prevalence and incidence rates. Therefore, we decided not to indicate the 95% CIs for the prevalence and incidence values throughout the text. All statistical analyses were performed using SAS software, version 9.2 (SAS Institute, Cary, NC, USA).

### 2.7. Statement of Ethics

Access to the dataset was strictly restricted to researchers authorized by HIRA. The study was conducted according to the guidelines of the Declaration of Helsinki, and approved by the Institutional Review Board (IRB) of the Catholic University of Korea (PC22ZISI0086, 6 May 2022). The requirement for informed consent was waived because all data were fully anonymized and de-identified by HIRA prior to the analysis.

## 3. Results

### 3.1. Study Population Selection

From the initial screening, 115,906 female patients with a diagnostic claim for hyperprolactinemia (ICD-10: E221) were identified. After applying the exclusion criteria—removing 19,010 patients during the 2007–2008 look-back period, 85 patients with Parkinson’s disease, and 1195 patients aged 9 years or younger and 60 years or older—a final cohort of 95,616 patients aged 10 to 59 years was established for longitudinal analysis.

### 3.2. Prevalence of Hyperprolactinemia

**Figure 1:**
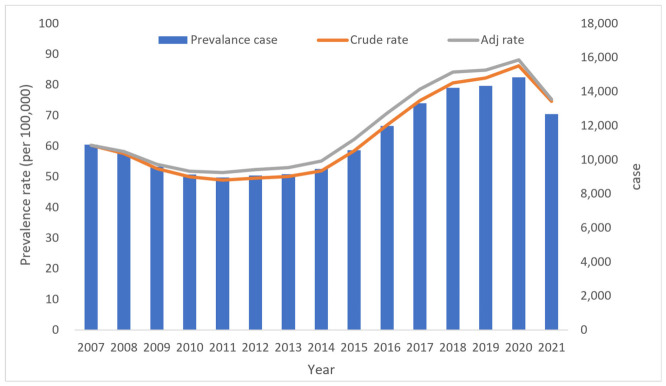
The crude prevalence rate (orange) and age-adjusted prevalence rate (grey) of patients with hyperprolactinemia in Korea, 2007–2021 by year.

The total number of treated patients continuously increased from 10,895 in 2007 to a peak of 14,848 in 2020, but showed a slight decreasing trend to 12,672 in 2021 (Figure 1). Table 1 shows the crude prevalence rate by age group from 2007 to 2021. The overall crude prevalence rate increased steadily from 60 per 100,000 persons in 2007 to 86 per 100,000 persons in 2020, and was recorded at 75 per 100,000 persons in 2021. The age-adjusted prevalence rate exhibited a similar pattern, starting at 60 per 100,000 persons in 2007, rising to 88 per 100,000 persons in 2020, and dropping to 75 per 100,000 persons in 2021.

**Figure 2:**
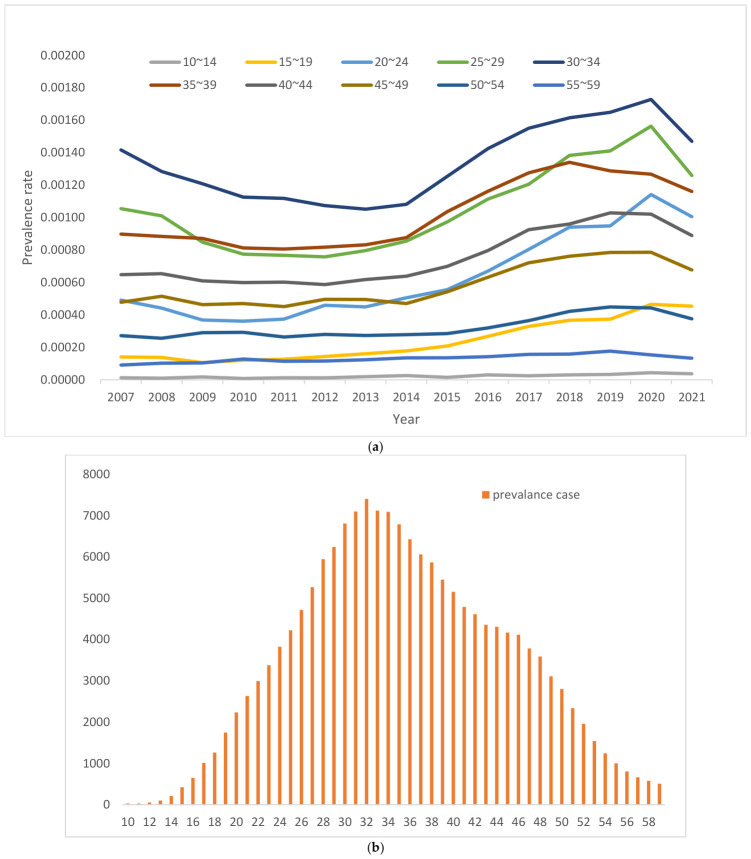
(**a**) Age-specific prevalence rates of hyperprolactinemia, 2007–2021. (**b**) Peak age of prevalence.

Age-specific analysis revealed that the prevalence rate increased gradually through the teens and early twenties, and showed a sharp upward curve starting in the late twenties (25–29 years) (Figure 2a). Specifically, the number of patients surged from 4223 at age 25 to 6242 at age 29, and the highest concentration of patients was observed in the subsequent early thirties (30–34 years) age group. The absolute peak in prevalence was recorded among women age 32 years, reaching a total of 7405 patients (Figure 2b). Following this peak, a steady and distinct declining trend was observed as age increased, dropping to 5154 patients at age 40, 2802 at age 50, and eventually falling to 507 patients at age 59.

### 3.3. Incidence of Hyperprolactinemia

**Figure 3:**
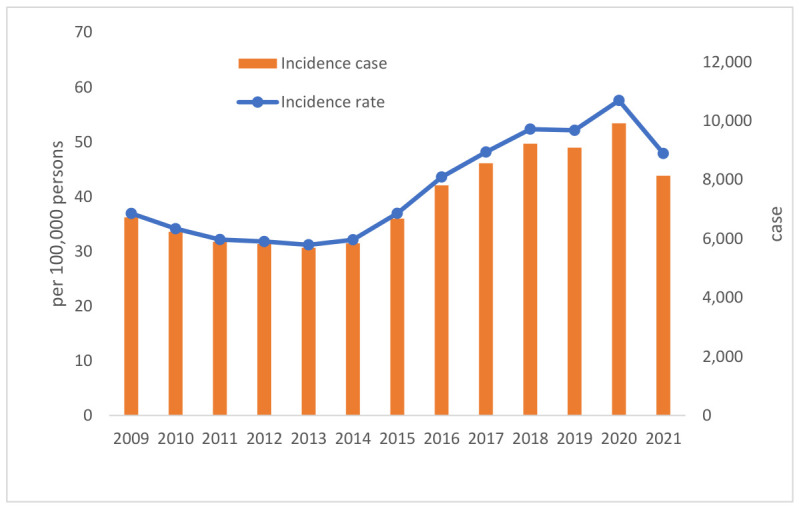
Annual incidence case (orange) and incidence rate (blue) of hyperprolactinemia, 2009–2021.

Analysis of the incidence data over the 13-year period from 2009 to 2021 identified a total of 95,616 newly diagnosed cases of hyperprolactinemia. The annual number of incident cases initially showed a decreasing trend from 6724 cases in 2009 to 5692 cases in 2013. However, the incidence gradually rebounded and steadily climbed thereafter, reaching a peak of 9914 newly diagnosed cases in 2020, followed by 8137 incident cases in 2021 (Figure 3).

**Figure 4:**
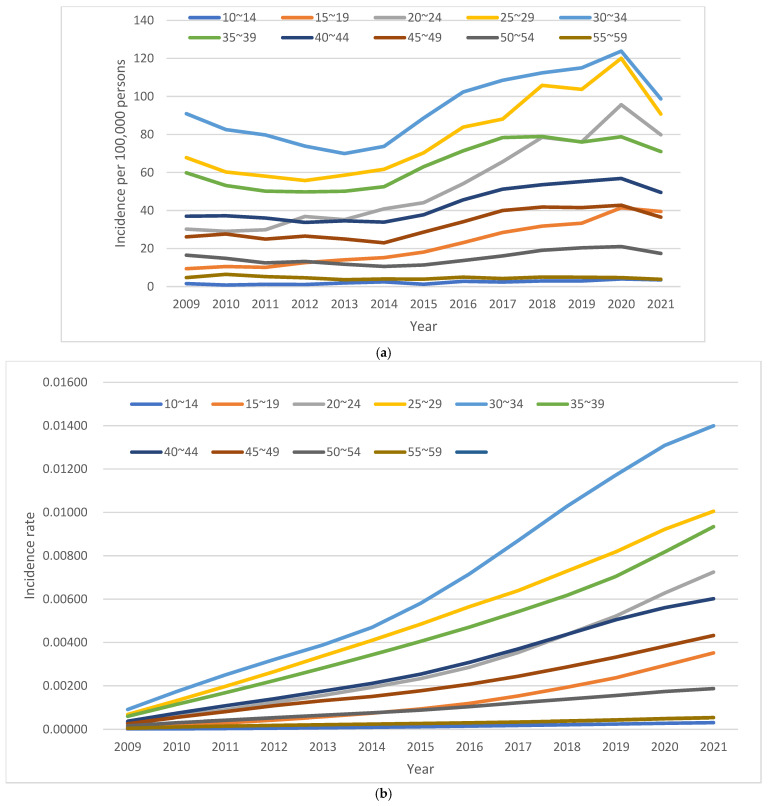
(**a**) Annual incidence rate for hyperprolactinemia by age group, 2009–2021. (**b**) Cumulative incidence rate for hyperprolactinemia by age group, 2009–2021.

Age-specific incidence analysis corroborated the prevalence patterns, demonstrating that the onset of the disease was highly concentrated among women of childbearing age. The 30–34 age group accounted for the largest proportion of all incident cases with 21,220 newly diagnosed patients (22.2%), followed by the 25–29 age group (16,743 cases, 17.5%) and the 35–39 age group (16,385 cases, 17.1%) (Figure 4).

### 3.4. Diagnostic and Cardiovascular Safety Surveillance Patterns

During the study period, for etiological diagnostic evaluations, a total of 5076 patients (5.3%) underwent sella MRI, while sella cone view radiography was performed in 1468 patients (1.5%). For cardiovascular safety and complication surveillance, claims for ECG were documented in 34,589 patients (36.2%), and echocardiography was performed in 505 patients (0.5%) among the total cohort.

### 3.5. Pharmacological Treatment Patterns

Among the 95,616 cases of hyperprolactinemia, pharmacological treatment with dopamine agonists was initiated in a substantial proportion of patients (44.5%, *n* = 42,503). Bromocriptine was the most frequently prescribed medication, administered in 25.75% of the patients (*n* = 24,622), while cabergoline was prescribed to 18.7% (*n* = 17,881). The age-specific prescription distribution revealed that both medications were most frequently prescribed to patients in their early thirties (30–34 years), which coincides with the peak prevalence age group. Specifically, within this age group, bromocriptine and cabergoline were prescribed in 6550 and 4562 cases, respectively, marking the highest prescription rate across all age brackets.

**Figure 5:**
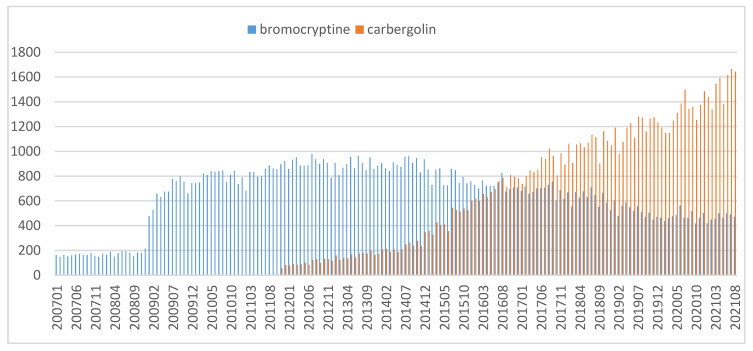
Annual trends in the prescription of dopamine agonists (bromocriptine vs. cabergoline), 2009–2021.

A longitudinal analysis of annual prescription volumes showed a gradual change in the choice of dopamine agonists (Figure 5). In the early observation period, bromocriptine was predominantly prescribed, with the annual prescription volume increasing to 10,812 cases in 2014. However, cabergoline prescriptions began to increase steadily after its initial recording in 2011 (136 cases), reaching 5524 cases in 2015. A crossover in prescription frequency occurred between 2016 and 2017; by 2017, cabergoline (10,619 cases) surpassed bromocriptine (8217 cases). This trend continued until the end of the study period in 2021, at which point cabergoline was prescribed in 12,218 cases compared to 3745 cases for bromocriptine.

### 3.6. Associated Clinical Conditions and Temporal Associations

A comprehensive analysis of associated clinical conditions was conducted, differentiating between the overall prevalence of associated diseases and their new incidence occurring subsequent to the diagnosis of hyperprolactinemia (Supplemental Table S1).

- Endocrine and Metabolic Disorders: Dyslipidemia was the most prevalent associated condition overall, affecting 55.8% (*n* = 53,337) of the cohort, with 24.8% (*n* = 23,758) of these cases developing after the initial diagnosis of hyperprolactinemia. Thyroid disease was also highly prevalent, observed in 48.4% (*n* = 46,315) of patients, with a post-diagnosis incidence of 19.9% (*n* = 19,005). Furthermore, diabetes mellitus (DM) and hypertension demonstrated a prevalence of 23.7% (*n* = 22,656) and 15.4% (*n* = 14,759), respectively, and a post-diagnosis incidence of 12.7% (*n* = 12,107) and 7.5% (*n* = 7147), respectively.
- Gynecological and Reproductive Disorders: Infertility exhibited a high overall prevalence of 28.0% (*n* = 26,738), but a relatively low incidence of 6.3% (*n* = 6062) developing after the diagnosis of hyperprolactinemia. Similarly, polycystic ovarian syndrome (PCOS) had a notable prevalence of 24.8% (*n* = 23,740), accompanied by a post-diagnosis incidence rate of 6.7% (*n* = 6422).
- Bone Disorders: Osteoporosis and osteopenia were identified in 13.1% (*n* = 12,490) and 3.3% (*n* = 3318) of the patients, respectively. The rates of incidence developing after the hyperprolactinemia diagnosis were 8.2% (*n* = 7799) for osteoporosis and 2.0% (*n* = 1892) for osteopenia.
- Malignancy: Ovarian (1.4%, *n* = 1364) and breast (1.2%, *n* = 1132) cancers were the most common malignancies. A substantial majority of breast cancer (0.9%, *n* = 876) developed after the diagnosis of hyperprolactinemia.

## 4. Discussion

This nationwide, population-based retrospective cohort study provides a comprehensive overview of the epidemiological characteristics, longitudinal shifts in pharmacological treatments, and the temporal dynamics of clinical conditions associated with hyperprolactinemia in Korean women. By utilizing an extensive 13-year dataset, the study captures real-world clinical trajectories and highlights critical shifts in disease management.

The epidemiological data reveal that the prevalence and incidence of hyperprolactinemia are overwhelmingly concentrated among women of childbearing age, particularly peaking in the early thirties (absolute peak at age 32). These findings are highly consistent with previous population-based studies, which reported that the prevalence of prolactinomas in females was highest in the 25–34 age group [30,31]. This pronounced diagnostic concentration between ages 26 and 32 is driven by dual factors. Biologically, this period represents the physiological zenith of endogenous estrogen production, a hormone known to directly stimulate lactotroph cell proliferation and upregulate prolactin gene transcription [5,32]. Clinically, it represents the peak window for female healthcare-seeking behavior related to family planning, infertility investigations, and medical concerns (e.g., amenorrhea, oligomenorrhea) [7,33]. The steady increase in disease prevalence and incidence up to 2020 indicates enhanced diagnostic awareness and increased healthcare access. The slight decline observed in 2021 is presumed to be a consequence of the prolonged COVID-19 pandemic, which likely led to a reduction in hospital visits and screening tests, rather than a true decrease in the disease burden [34].

A pivotal finding of this study is the distinct paradigm shift in the pharmacological management of hyperprolactinemia. Our longitudinal analysis demonstrates a definitive crossover between 2016 and 2017, where cabergoline prescription volumes surpassed those of bromocriptine. By 2021, cabergoline had firmly established itself as the dominant primary therapy. This transition is intrinsically linked to the historical changes in drug accessibility in South Korea. Prior to 2015, cabergoline was not commercially distributed by local pharmaceutical companies and was only available through the Korea Orphan & Essential Drug Center, which severely limited its clinical use. The formal inclusion of cabergoline in the national health insurance coverage in January 2015 marked a pivotal turning point in its clinical adoption, significantly enhancing its accessibility. This policy change enabled clinicians to fully leverage cabergoline’s superior efficacy in normalizing serum prolactin levels, alongside its highly favorable tolerability profile and convenient weekly dosing schedule [7,15].

A key finding reflecting real-world clinical practice in Korea is that only 5.3% of the entire cohort underwent a sella MRI. In highly controlled clinical guidelines, neuroimaging is strongly recommended to differentiate organic prolactinomas from non-tumor etiologies [7]. However, our findings reflect a distinct real-world pattern where a vast majority of claims-defined cases are managed in primary or secondary care settings as functional, transient, or drug-induced conditions, without escalating to high-cost diagnostic imaging. This structural heterogeneity is a primary characteristic of population-level claims data, and the pattern aligns with large-scale European epidemiological registries, such as the PROLEARS study in Scotland [35], which demonstrated that a substantial portion of hyperprolactinemia cases in the general population are transient or drug-induced rather than verified organic adenomas.

The stratification of associated clinical conditions into overall prevalence and post-diagnosis incidence yields profound clinical insights. Gynecological and reproductive conditions, notably infertility and PCOS, exhibited high overall prevalence but a markedly low subsequent incidence. The general prevalence of infertility in the South Korean female population is estimated to be approximately 13.5% [36], which aligns with the global estimate of 1 in 6 individuals (approximately 15%) reported by the World Health Organization (WHO) [37]. Similarly, recent nationwide studies report the age-adjusted prevalence of PCOS among Korean women to be approximately 4.3% [38], which is lower than the global estimate of 10–13% [39]. Our hyperprolactinemia cohort demonstrated exceptionally high overall prevalence for these conditions (28.0% for infertility and 24.8% for PCOS). This pronounced discrepancy from the general population, coupled with their notably low post-diagnosis incidence, strongly suggests that hyperprolactinemia and these reproductive disorders are frequently evaluated and diagnosed concurrently during the initial work-up for menstrual irregularities or infertility. Rather than hyperprolactinemia acting solely as a downstream cause, these conditions share complex, overlapping endocrine pathways that prompt simultaneous identification [10,18,40]. This finding is further supported by Luque-Ramirez et al. [41], who highlighted that mild hyperprolactinemia is a common feature in women with PCOS.

In contrast, among patients who developed bone disorders, the vast majority of cases occurred subsequent to the hyperprolactinemia diagnosis. Specifically, 62.4% of all osteoporosis cases (*n* = 7799 out of 12,490) and 60.3% of all osteopenia cases (*n* = 1892 out of 3138) were newly documented after the index hyperprolactinemia claim. Patho-physiologically, hyperprolactinemia inhibits hypothalamic GnRH secretion, leading to a state of secondary hypogonadism and chronic estrogen deficiency [1,42]. This hormonal imbalance disrupts the bone remodeling equilibrium, favoring bone resorption over formation and ultimately resulting in bone mineral density loss [25]. Our findings support the necessity of early and regular bone mineral density (BMD) monitoring, a clinical imperative also emphasized by Naliato et al., who demonstrated that significant bone density deficits can persist in women with prolactinoma even after the initiation of dopamine agonist therapy [43]. Furthermore, Bussade et al. [44] reported that a substantial reduction in BMD occurs even in premenopausal women with prolactinoma, highlighting that the hypogonadal environment associated with elevated prolactin poses a severe skeletal risk regardless of the patient’s age or menopausal status. Consequently, our data reinforce the importance of bone health management as a standard component of long-term care for all women with hyperprolactinemia.

Substantial proportions of diabetes mellitus (53.44%) and hypertension (48.42%) cases were also newly diagnosed subsequent to the onset of hyperprolactinemia. Considering the potential impact of prolactin-induced insulin resistance or prolonged hormonal imbalance on metabolic syndrome [45,46], prophylactic monitoring of metabolic parameters should be incorporated into the routine management of these patients.

This study has several limitations inherent to the use of administrative claims data. First, exact clinical parameters, including serum prolactin concentration levels, specific pituitary tumor dimensions (microadenoma vs. macroadenoma), and body mass index (BMI), were inaccessible. Consequently, a definitive biochemical or radiological differentiation between pure organic prolactinomas, drug-induced hyperprolactinemia, and rare overlapping conditions like growth hormone-secreting somatotropinomas could not be executed. While patients with Parkinson’s disease were excluded to filter out high-dose dopamine agonist users, the clinical heterogeneity within the broad E221 claim code remains an inherent constraint in etiological subgrouping. Furthermore, actual patient adherence to the prescribed dopamine agonists cannot be definitively confirmed through prescription claims alone. Additionally, while our study captured the 2017 nationwide shift from bromocriptine to cabergoline, individual dose titrations and drug-switching patterns were not analyzed. These population-level trends reflect overall healthcare utilization rather than personalized therapeutic scaling or patient compliance.

Second, as the HIRA database consists of administrative claims data primarily designed for billing and reimbursement, it lacks the level of detail found in specialized clinical research databases. Since diagnoses are defined based on ICD-10 codes, discrepancies may exist between billed claims and actual clinical diagnoses, potentially introducing misclassification bias. Furthermore, the true burden of the disease may be underestimated, as patients with mild symptoms or those who did not seek medical care are not captured in the database.

Third, it is possible that our operational definition, which relied solely on diagnostic codes without requiring a minimum number of medical visits (e.g., at least one hospitalization or two outpatient visits) or the presence of pharmacological treatment, may have led to an overestimation of the patient count. However, we deliberately avoided visit-based restrictions to prevent selection bias, which can occur by excluding subjects at the temporal boundaries of the cohort window. Moreover, since pharmacological therapy is not clinically mandatory for all hyperprolactinemia cases depending on the patient’s condition, its presence was not required for inclusion in the study cohort.

Despite these limitations, the strength of our study lies in its massive, nationwide cohort and the precise temporal delineation of associated clinical conditions. The findings clearly illustrate a modernized landscape of hyperprolactinemia treatment and strongly demonstrates the need for a multidisciplinary clinical approach. Recognizing the high post-diagnosis rate of bone and metabolic disorders will enable clinicians to optimize preventative care, thereby improving the long-term quality of life for women diagnosed with hyperprolactinemia.

## 5. Conclusions

This 13-year nationwide study reveals that hyperprolactinemia in Korean women peaks during the early thirties, significantly impacting reproductive health. We identified a major pharmacological shift from bromocriptine to cabergoline, primarily driven by the 2015 expansion of national health insurance coverage. Crucially while PCOS and infertility typically diagnosed concurrently with hyperprolactinemia, bone loss and metabolic complications frequently emerge as long-term sequelae. Therefore, clinical management must extend beyond prolactin normalization to include proactive, multidisciplinary screening for skeletal and metabolic health to improve long-term quality of life.

## Figures and Tables

**Table 1:** The crude prevalence rate, 2007–2021.

	10~14 Years			15~19 Years			20~24 Years			25~29 Years			30~34 Years			35~39 Years			40~44 Years			45~49 Years			50~54 Years			55~59 Years		
	Case (Per 100,000)		Population	Case (Per 100,000)		Population	Case (Per 100,000)		Population	Case (Per 100,000)		Population	Case (Per 100,000)		Population	Case (Per 100,000)		Population	Case (Per 100,000)		Population	Case (Per 100,000)		Population	Case (Per 100,000)		Population	Case (Per 100,000)		Population
2007	21	1	1,653,961	216	14	1,539,263	802	49	1,634,016	2065	105	1,957,996	2877	142	2,030,319	2023	90	2,253,058	1315	65	2,029,760	1020	48	2,134,888	446	27	1,640,953	110	9	1,209,075
2008	16	1	1,631,851	218	14	1,579,662	684	44	1,548,865	1995	101	1,973,735	2514	128	1,958,242	1984	88	2,246,169	1353	65	2,068,594	1110	51	2,156,501	445	26	1,738,721	128	10	1,250,799
2009	28	2	1,600,103	171	11	1,614,483	555	37	1,505,980	1645	85	1,938,744	2303	121	1,905,463	1934	87	2,220,229	1298	61	2,129,227	994	46	2,146,753	538	29	1,854,222	135	10	1,299,880
2010	13	1	1,567,231	199	12	1,648,470	538	36	1,489,000	1431	77	1,847,775	2149	113	1,908,572	1764	81	2,171,434	1297	60	2,165,754	994	47	2,115,794	573	29	1,958,329	179	13	1,398,967
2011	19	1	1,524,601	211	13	1,662,199	561	37	1,499,241	1340	77	1,746,765	2165	112	1,936,026	1691	81	2,096,955	1333	60	2,213,982	930	45	2,060,008	542	26	2,055,638	174	11	1,527,939
2012	17	1	1,452,667	236	14	1,649,308	707	46	1,538,723	1245	76	1,642,257	2107	107	1,962,313	1658	82	2,027,689	1320	59	2,248,909	1005	50	2,026,583	596	28	2,128,758	189	12	1,631,898
2013	26	2	1,375,262	261	16	1,627,421	709	45	1,578,376	1242	80	1,558,881	2081	105	1,978,039	1627	83	1,955,181	1385	62	2,240,479	1021	49	2,063,381	587	27	2,148,726	213	12	1,728,364
2014	34	3	1,307,481	284	18	1,596,576	815	51	1,613,095	1297	85	1,517,074	2102	108	1,943,364	1668	88	1,902,830	1414	64	2,213,903	997	47	2,122,496	594	28	2,137,493	249	14	1,842,154
2015	18	1	1,226,236	326	21	1,564,661	915	56	1,647,071	1459	97	1,499,816	2323	125	1,853,636	1976	104	1,906,997	1513	70	2,165,573	1170	54	2,158,416	600	28	2,106,345	264	14	1,945,185
2016	35	3	1,153,484	407	27	1,522,737	1,112	67	1,660,504	1680	111	1,508,716	2498	142	1,753,196	2248	116	1,935,025	1666	80	2,091,518	1394	63	2,206,120	656	32	2,050,850	290	14	2,041,486
2017	28	2	1,130,276	478	33	1,451,429	1,324	80	1,647,663	1864	120	1,546,920	2557	155	1,648,854	2502	128	1,961,526	1871	93	2,022,653	1616	72	2,241,024	736	36	2,017,624	331	16	2,114,005
2018	34	3	1,127,731	504	37	1,374,316	1,529	94	1,625,826	2193	138	1,585,850	2529	162	1,565,828	2651	134	1,977,219	1873	96	1,950,445	1700	76	2,232,481	866	42	2,054,134	338	16	2,134,289
2019	37	3	1,117,638	488	37	1,306,357	1,512	95	1,594,617	2285	141	1,619,563	2512	165	1,523,573	2501	129	1,941,960	1953	103	1,897,420	1730	78	2,204,698	948	45	2,111,877	376	18	2,122,997
2020	49	4	1,122,008	570	47	1,225,107	1,785	114	1,562,539	2584	156	1,652,449	2604	173	1,506,688	2348	127	1,852,902	1941	102	1,901,418	1695	79	2,156,068	951	44	2,147,074	321	15	2,092,130
2021	42	4	1,139,096	523	45	1,152,557	1,528	100	1,520,709	2096	126	1,665,042	2228	147	1,515,957	2036	116	1,753,790	1716	89	1,929,716	1409	68	2,082,435	824	38	2,194,596	270	13	2,037,429
total	417	32	20,129,626	5092	359	22,514,546	15,076	951	23,666,225	26,421	1575	25,261,583	35,549	2005	26,990,070	30,611	1534	30,202,964	23,248	1129	31,269,351	18,785	873	32,107,646	9902	485	30,345,340	3567	197	26,376,597

## Data Availability

The raw data supporting the conclusions of this article will be made available by the authors on request.

## Supplementary Materials

The following supporting information can be downloaded at https://www.mdpi.com/article/10.3390/jcm15124411/s1: Table S1: Prevalence and post-diagnosis incidence of comorbidities in women with hyperprolactinemia.
