# Supplementary material for: Incidence, Treatment Patterns, and Associated Clinical Conditions of Hyperprolactinemia Identified via Nationwide Claims Data in Korea: A 13-Year Population-Based Study
Source: J Clin Med. 2026 Jun 7;15(12):4411. doi: 10.3390/jcm15124411 (PMC13301055; doi:10.3390/jcm15124411)
Supplement: Supplementary file 1 [file jcm-15-04411-s001.zip › jcm-4347212-supplementary.pdf]

Supplemental Table S1. Prevalence and post-diagnosis incidence of comorbidities in women with hyperprolactinemia (n=95,616)

Category	Comorbidity	Overall prevalence, n (%)	Post-diagnosis incidence, n (%)
Endocrine & Metabolic	Dyslipidemia	53,337 (55.8)	23,758 (24.8)
	Thyroid disease	46,315 (48.4)	19,005 (19.9)
	Diabetes mellitus	22,656 (23.7)	12,107 (12.7)
	Hypertension	14,759 (15.4)	7,147 (7.5)
	Obesity	3,639 (3.8)	1,634 (1.7)
Gynecological	Infertility	26,738 (28)	6,062 (6.3)
	Polycystic ovary syndrome	23,740 (24.8)	6,422 (6.7)
Bone Disorders	Osteoporosis	12,490 (13.1)	7,799 (8.2)
	Osteopenia	3,138 (3.3)	1,892 (2)
Malignancies	Ovarian cancer	1,364 (1.4)	724 (0.8)
	Breast cancer	1,132 (1.2)	876 (0.9)
	Endometrial cancer	449 (0.5)	286 (0.3)
	Cervical cancer	330 (0.3)	160 (0.2)
	Other gynecological cancers	93 (0.1)	48 (0.05)
	Vulvar cancer	8 (0.01)	4 (0.004)
	Vaginal cancer	4 (0.004)	3 (0.003)
